# Impact of COVID-19 and Shelter in Place on Volume and Type of Traumatic Injuries

**DOI:** 10.5811/westjem.2021.5.49968

**Published:** 2021-09-02

**Authors:** James Murrett, Emily Fu, Zoe Maher, Crystal Bae, Wayne A. Satz, Kraftin E. Schreyer

**Affiliations:** * Temple University Hospital, Department of Emergency Medicine, Philadelphia, Pennsylvania; † Temple University Hospital, Department of Surgery, Philadelphia, Pennsylvania

## Abstract

**Introduction:**

Very little is known about the effects of the novel coronavirus (COVID-19) pandemic and its associated social distancing practices on trauma presentations to the emergency department (ED). This study aims to assess the impact of a city-wide stay at home order on the volume, type, and outcomes of traumatic injuries at urban EDs.

**Methods:**

The study was a retrospective chart review of all patients who presented to the ED of an urban Level I Trauma Center and its urban community affiliate in the time period during the 30 days before the institution of city-wide shelter-in-place (preSIP) order and 60 days after the shelter-in-place (SIP) order and the date-matched time periods in the preceding year. Volume and mechanism of traumatic injuries were compared using paired T-tests.

**Results:**

There was a significant decrease in overall ED volume. The volume of certain blunt trauma presentations (motor vehicle collisions) during the first 60 days of SIP compared to the same period from the year prior also significantly decreased. Importantly, the volume of penetrating injuries, including gunshot wounds and stab wounds, did not differ for the preSIP and SIP periods when compared to the prior year. The mortality of traumatic injuries was also unchanged during the SIP comparison period.

**Conclusion:**

While there were significant decreases in visits to the ED and overall trauma volume, penetrating trauma, including gun violence, and other severe traumatic injuries remain a public health crisis that affects urban communities despite social distancing recommendations enacted during the COVID-19 pandemic.

## INTRODUCTION

The novel coronavirus 2019 (COVID-19) pandemic has drastically changed healthcare utilization in the United States, in part, through a decreased presentation of many medical conditions. A poll by the American College of Emergency Physicians indicated that 29% of adults have “actively delayed or avoided seeking medical care due to concerns about contracting COVID-19.” 1 Public avoidance of EDs in the face of critical illness is reflected in a decreased number of interventions. For example, a study of high-volume cardiac catheterization labs across the US showed a 38% reduction in ST-elevation myocardial infarction activations in March 2020 compared to prior months 2 and neuroimaging for strokes decreased by 39% across 856 hospitals in all states.^3^ The pandemic’s impact on medical presentations to EDs in the US mirrors international trends. Reports from Canada, 4 China,^5^ and Italy^6^ show reduced presentations of stroke, myocardial infarction, and acute heart failure, respectively.

The decline in ED volume secondary to decreased presentations of non-COVID-19 medical illness is a widely acknowledged phenomenon, but less is known about the pandemic’s effect on patients with traumatic injuries. Studies at a large urban Italian hospital^7^ and a trauma center in New Zealand^8^ showed a 20% and 43% decline in trauma-related cases and injury-related admissions, respectively. Both studies attributed this to a decline in vehicular injuries, which had previously constituted a large part of their trauma census. This decline has also been reflected in data from US trauma centers. An early study of two Santa Clara Level I Trauma Centers showed a 4.8-fold reduction in trauma activations compared to month-matched cohorts from prior years, of which the majority were due to vehicular trauma.^9^ A Level II trauma center in New Hampshire similarly showed a 57.4% decrease in trauma admissions, largely driven by an 80% decrease in motor vehicle collisions (MVCs).^10^

However, the effects of the COVID-19 pandemic and subsequent stay-at-home orders on penetrating trauma are developing. In Philadelphia, an initial analysis of the first six weeks of the pandemic revealed a 20% decrease in trauma contacts compared to the prior year with a significant increase in the number and proportion of penetrating trauma cases.^11^ It cannot be extrapolated from international data, as the US as a whole carries a higher burden of gun violence than most other countries. In 2018, firearm homicide was the fifth leading cause of death in all age groups in the US with 13,957 deaths^12^; and in 2016 the US accounted for 35% of global firearm suicides with 22,936 deaths and 9% of global firearm homicides with 14,414 deaths.^13^

While it makes intuitive sense that restrictions on travel and commuting would decrease blunt trauma volumes, the effects of social distancing on penetrating trauma and specifically gun-related injury are much less predictable. Trends in trauma volumes vary largely across American cities. Gun violence, for example, increased 11.7% in New York City from January to April 2020, 2% in Baltimore, and 23% in Chicago, while it decreased 9.3% in Los Angeles^14^ compared to prior years. Nationwide trends have been concerning: March 2020, the month in which 30 states instituted stay-at-home orders,^15^ was also the second busiest month for gun sales in US history.^16^ At the same time, media sites in cities across the US including Detroit,^17^ Louisville,^18^ Chicago,^19^ and Philadelphia^20^ reported increases in shootings even after shelter-in-place (SIP) injunctions.

However, there has been no published data on the consequences of stay-at-home orders on presentation of penetrating trauma to EDs in the US. Our goal in this study was to assess the impact of COVID-19 SIP orders on the volume of various categories of blunt and penetrating trauma at two urban EDs: a Level I trauma center, and a community affiliate.

**Population Health Research Capsule**

What do we already know about this issue?

*During the early stages of the coronavirus disease 2019 (COVID-19) pandemic, there was a decline in emergency department (ED) presentations of many medical illnessesconditions, but less is known about traumatic injuries*.

What was the research question?

*To assess the impact of a citywide stay-at-home order on traumatic injuries at urban EDs*.

What was the major finding of the study?

*While there was a significant decrease in overall ED volume, the volume of penetrating injuries did not change*

How does this improve population health?

*Penetrating trauma, including gun violence, remains a public health crisis despite social distancing recommendations enacted during the COVID-19 pandemic*.

## METHODS

We conducted a retrospective, observational study of all patients who presented to the EDs of Study Site A and its community academic affiliate, Study Site B. Study Site A is a 50-bed ED at an urban, academic, Level I trauma center in an area with high rates of gun violence.^21^ Study Site B is a 19-bed urban, community-affiliate ED. Combined, these two sites have an annual volume of 140,000 visits and 2,700 trauma activations.

We extracted all data from our electronic health record (EHR) (Epic Systems Corporation, Verona, WI). Included patients were those who presented with traumatic injuries and were identified by searching the diagnosis field of the EHR for specific text strings representing trauma diagnoses including, “gun shot,” “stab wound,” “motor vehicle collision,” “pedestrian,” “assault,” and “fall.” These diagnoses are manually entered by emergency or trauma surgery providers at time of disposition and are not mutually exclusive.

The charts from the study periods were selected for analysis as seen in the Figure. Our study period included the 30 days prior to the SIP order (preSIP) and the first 60 days of the SIP order, which went into effect in Philadelphia on March 23, 2020. The order prohibited all public and private gatherings, limited in-person work at all but essential businesses, and discouraged leaving personal residences excepting in the case of life-sustaining activities.^22^ The PreSIP time period from February 22–March 22, 2020 was compared to a 30-day period in 2019 from February 21–March 22, 2019. The first 60 days of the SIP order were divided into the first 30 days from March 23–April 21, 2020 and the second 30 days from April 22–May 21, 2020 and were compared to the same dates from 2019. The daily frequency and type of trauma activations and patients presenting to the ED who received a diagnosis consistent with a traumatic injury in different time periods were then compared year-to-year using two-sample t-tests. By convention, significance was set at *P* <0.05. This study was approved by the study institution’s institutional review board.

## RESULTS

### Total Emergency Department Visits

There were 11,979 visits in the preSIP period in 2020 compared to 11,956 for the 2019 comparison period. This difference was not significant. There were 7,948 visits during the first 30 days of the SIP period and 8,379 visits during the second 30 days for the SIP period compared to 12,002 and 12,040 for the same periods of time in 2019. This represents a 32% decrease in ED volumes for the first 60 days of SIP. The difference between volumes in all the SIP periods (first 30 days, second 30 days, and first 60 days) were significant compared to 2019 (*P* <0.05).

Summary data for trauma activations and traumatic injuries are shown in Table 1. The volume of trauma activations for the preSIP was higher in 2020 than in 2019. There were 232 trauma activations during the preSIP period compared to 179 in the first 30 days and 231 in the second 30 days of SIP. In 2019 the number of traumas increased from 183 in the 30 days prior to March 23 to 234 in the 30 days after and 219 in the subsequent 30 days. There was a significant difference in the number of trauma activations from year-to-year for the preSIP period compared to 2019 (*P* = 0.04). While the number of trauma activations was significantly different for the first 30 days of SIP compared to 2019 (*P* = 0.01), the differences were not significant for the second 30 days or considering the full first 60 days of SIP.

Demographic data for all traumatic injuries are shown in Table 2. The patients presenting with traumatic injuries in 2020 were, on average, two years older and more often male. The acuity and mortality were slightly higher in 2020 compared to 2019, but these differences were not statistically significant.

Demographic data for penetrating injuries is shown in Table 3. Similar to the trends for all traumatic injuries, year-to-year trends are evident. There were, on average, 10 additional patients with traumatic injuries during 2020, compared to the same time periods in 2019. The patients presenting with traumatic injuries in 2020 were also, on average, older and more often male. Before the SIP order in 2020, 100% of patients with traumatic injuries were male. The first 30 days of SIP showed an average increase of three years in the age of patients presenting with gunshot wounds or stab wounds. While the acuity was similar year-to-year, the mortality demonstrated mixed trends.

## DISCUSSION

Although other etiologies of trauma and overall trauma volume decreased in the weeks following SIP orders, we found no significant change in the incidence of penetrating trauma pre- and post-SIP. However, the proportion of penetrating trauma increased during the COVID-19 pandemic, including during a SIP order, despite a decrease in overall ED volume. The persistent volumes of penetrating trauma during the novel COVID-19 pandemic reflect the fact that gun violence is a public health crisis with multifactorial contributors not reduced by simple social distancing policies. In fact, it is likely that the pandemic has exacerbated key drivers of this public health crisis. Prior studies have shown a strong correlation between penetrating trauma and unemployment^23^ and poverty rates.^24^ While there are many sociodemographic factors that contribute to rates of penetrating trauma, the role of the rising US unemployment rate during the COVID-19 pandemic to rates comparable to the Great Recession^25^ cannot be overlooked.

Regional gun homicide rates also correlate with income inequality, level of citizens’ trust in institutions, poverty levels, and concentrations of vacant housing.^24^ During the COVID-19 pandemic, the US wealth gap has widened,^26^ and one in three Americans reported inability to pay rent in April 2020, with up to 10% of those polled facing eviction.^27^ The above study also notes that gun homicide rates reflect socioeconomic determinants with a lag time of up to 17 years. Considering this, the socioeconomic effects of the coronavirus on gun violence bear further longitudinal study.

In addition to the pandemic’s effect on socioeconomic drivers of gun violence, there is also an intangible element of emotional stress. Experts warn that the social isolation, anxiety, and fear caused by the disease are contributing to an unprecedented national mental health crisis, with a third of Americans displaying clinical signs of anxiety or depression since the pandemic began.^28^ Those under financial duress are more likely to have both mental health issues and a lack of access to resources such as counseling or psychiatric care. One research institute predicts that this “perfect storm” of risk factors could lead to a 20–30% increase in firearm suicides based on rising unemployment rates.^29^

Many of the above factors likely contribute to the persistent high volume of gun trauma that we have reported in this study. Furthermore, they have a disproportionate effect on communities of color. Even prior to the economic downturn caused by the COVID-19 pandemic, the 2018 unemployment rate in the three districts surrounding Temple University Hospital Main Campus and Temple University Hospital Episcopal Campus was 18–23%, 14% above the national average. Within the study site service area, 26% of people live below the poverty line and 67.6% have a household income under $50,000.^30^

It is also notable that the service area of the two study sites is 75.9% Black and Latinx residents. Black and Latinx Americans have had significantly higher unemployment rates than the general public during the pandemic,^31^ are more likely to report housing insecurity,^32^ and less likely to use mental health resources.^33^ Historically these two populations also suffer a disproportionate share of gun homicides.^34^ A recent Pennsylvania Department of Health study showed a 27.3% firearm homicide rate among Black residents of the city and a 62.7% firearm mortality rate among Black males.^35^ As these rates reflect violent crime prior to the pandemic, it is likely that the socioeconomic consequences of COVID-19 have only compounded the pre-existing racial disparities in penetrating trauma.

Our findings for blunt trauma diagnoses are similar to those previously reported.^7^–^10^ The significant reduction in pedestrian accidents and MVCs is likely an indirect result of the SIP order, as fewer people were leaving their homes to walk or drive. The significant reduction in falls may also have been affected by reduced foot traffic during this time. While more study is required to understand the key influencers on changes in traumatic injury presentations to the ED during the novel coronavirus pandemic, it is clear that the pandemic’s disproportionate effect on key socioeconomic drivers of trauma bear further study.

## LIMITATIONS

There are several limitations to our study. These are observational data from a single center that may or may not represent general trends. These data do not support SIP orders as an independent predictor of changes in trauma-related ED visits. Diagnoses are entered by physicians at time of admission and discharge and may fail to reflect all patients who present with a specific trauma diagnosis. While trauma activations were included, they are defined according to hospital protocol with one component of the protocol allowing for physician discretion and, therefore, inherent provider-level variability. Furthermore, there is some subjectivity in the selection of a diagnosis for each patient and some patients may appear in multiple categories. For example, a patient may have diagnoses of both “assault” and “stab wound.” While these text strings represent the majority of mechanisms that resulted in trauma activations some text strings analyzed failed to reach a number worth analysis. For example, “motorcycle” revealed too few patients to include. Additionally, an analysis of “hemorrhage” resulted in non-trauma diagnoses and was not included.

## CONCLUSION

Although total ED volume decreased by one third after SIP orders were instituted as compared to the prior year, the volume of gunshot wounds and stab wounds did not significantly differ. While some categories of blunt trauma (motor vehicle collisions, falls) significantly decreased after SIP orders, both urban sites in this study continued to experience a similar volume of penetrating trauma despite restrictions on interpersonal gatherings and travel outside the home.

## Figures and Tables

**Figure:**
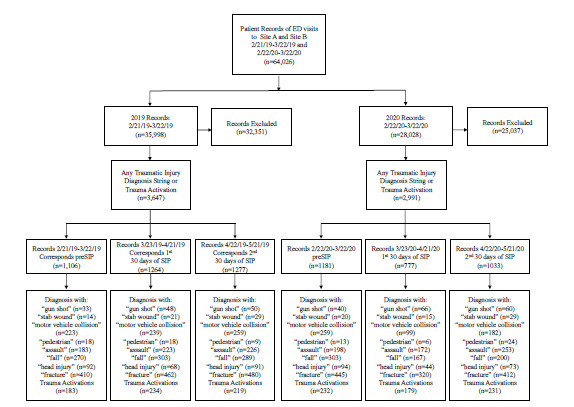
Patient records identified during data extraction and analysis process. *ED*, emergency department; *SIP*, shelter in place; *PreSIP*, pre-shelter in place.

**Table 1:** Traumatic injuries with percent change (2020 from 2019). Data reported as number with 95% confidence interval below, change reported as percent.

	Time period								

	Pre SIP			First 30 days SIP			Second 30 days SIP		
		
	2019	2020	Percent change	2019	2020	Percent change	2019	2021	Percent Change
Total ED visits	11,979 (11,522–12,435)	11,956 (11,349–12,563)	0%	12,002 (11,553–12,451)	7,948 (7,688–8,208)	−34%	12,040 (11,588–12,521)	8,379 (8,098–8660)	−30%
Trauma activations	183 (154–212)	232 (199–265)	27%	234 (204–264)	179 (149–209)	−24%	219 (185–253)	231 (202–260)	5%
GSW	33 (19–47)	40 (28–52)	21%	48 (34–62)	66 (49–83)	38%	50 (30–70)	60 (42–78)	20%
Stab	14 (6–22)	20 (12–28)	43%	21 (13–29)	15 (6–24)	−29%	29 (18–40)	29 (18–24)	0%
MVC	223 (183–263)	259 (218–300)	16%	239 (202–276)	99 (77–121)	−59%	259 (218–300)	182 (147–217)	−30%
Pedestrian	18 (10–26)	13 (6–20)	−28%	18 (9–27)	6 (1–11)	−67%	9 (3–14)	24 (13–35)	167%
Assault	183 (157–209)	198 (162–234)	8%	223 (194–252)	172 (145–199)	−23%	226 (194–258)	253 (217–289)	12%
Fall	270 (242–298)	303 (267–339)	12%	303 (264–342)	167 (144–190)	−45%	289 (262–316)	200 (173–227)	−31%

*ED*, emergency department; *PreSIP*, pre-shelter in place; *SIP*, shelter in place; *GSW*, gunshot wound; *MVC*, motor vehicle collision.

**Table 2:** Demographics of all injuries. Data reported as number; gender reported as percent (% male); age reported as mean; Emergency Severity Index (ESI) acuity reported as mean; and mortality reported as percent.

	Time period					

	PreSIP		First 30 days SIP		Second 30 days SIP	
		
	2019	2020	2019	2020	2019	2020
All injuries	724	790	825	497	831	703
Gender	51%	57%	56%	63%	54%	63%
Age	43.1	45.6	44.9	47.1	43.8	45.8
ESI acuity	2.98	2.95	2.88	2.84	2.96	2.81
Mortality	1.10%	1.14%	1.45%	2.82%	0.72%	1.71%

*SIP*, shelter in place.

**Table 3:** Demographics of patients presenting to emergency department with penetrating trauma (receiving diagnosis of “stab wound” or “gun shot”). Data reported as number (#), sex gender reported as percent (%) male, age reported as mean, Emergency Severity Index (ESI) acuity reported as mean, and mortality reported as (%).

	Time period					

	PreSIP		First 30 days SIP		Second 30 days SIP	
		
	2019	2020	2019	2020	2019	2020
Penetrating trauma	47	59	69	81	79	89
Gender	91%	100%	88%	88%	80%	87%
Age	34.0	35.5	35.0	42.1	34.6	36.6
ESI acuity	2.00	2.00	1.64	1.84	1.88	1.85
Mortality	8.51%	6.78%	11.59%	9.88%	3.80%	5.62%

*SIP*, shelter in place.

## References

[1] Emergency Physicians (2020). Public poll: emergency care concerns amidst COVID-19.

[2] Garcia S, Albaghdadi MS, Meraj PM (2020). Reduction in ST-segment elevation cardiac catheterization laboratory activations in the United States during COVID-19 pandemic. J Am Coll Cardiol.

[3] Kansagra AP, Goyal MS, Hamilton S (2020). Collateral effect of Covid-19 on stroke evaluation in the United States. N Engl J Med.

[4] Bullrich MB, Fridman S, Mandzia JL (2020). COVID-19: stroke admissions, emergency department visits, and prevention clinic referrals. Can J Neurol Sci.

[5] Tam C-CF, Cheung K-S, Lam S (2020). Impact of coronavirus disease 2019 (COVID-19) outbreak on ST-segment-elevation myocardial infarction care in Hong Kong, China. Circ Cardiovasc Qual Outcomes.

[6] Colivicchi F, Di Fusco SA, Magnanti M (2020). The impact of the coronavirus disease-2019 pandemic and Italian lockdown measures on clinical presentation and management of acute heart failure. J Card Fail.

[7] Comelli I, Scioscioli F, Cervellin G (2011). Impact of the COVID-19 epidemic on census, organization and activity of a large urban emergency department. Acta Bio-Medica Atenei Parm.

[8] Christey G, Amey J, Campbell A (2020). Variation in volumes and characteristics of trauma patients admitted to a level one trauma centre during national level 4 lockdown for COVID-19 in New Zealand. N Z Med J.

[9] Forrester JD, Liou R, Knowlton LM (2020). Impact of shelter-in-place order for COVID-19 on trauma activations: Santa Clara County, California, March 2020.

[10] Kamine TH, Rembisz A, Barron RJ (2020). Decrease in trauma admissions with COVID-19 pandemic. West J Emerg Med.

[11] Qasim Z, Sjoholm L, Volgraf J (2020). Trauma center activity and surge response during the early phase of the COVID-19 pandemic – the Philadelphia story. J Trauma Acute Care Surg.

[12] Centers for Disease Control and Prevention (2020). Leading Causes of Death and Injury – PDFs |Injury Center| CDC.

[13] Naghavi M, Marczak LB, Global Burden of Disease 2016 Injury Collaborators (2018). Global mortality from firearms, 1990–2016. JAMA.

[14] Sutherland M, McKenney M, Elkbuli A (2021). Gun violence during COVID-19 pandemic: paradoxical trends in New York City, Chicago, Los Angeles and Baltimore. Am J Emerg Med.

[15] Mervosh S, Lu D, Swales V (2020). See which states and cities have told residents to stay at home. The New York Times.

[16] Collins K, Yaffe-Bellany D (2020). About 2 million guns were sold in the US as virus fears spread. The New York Times.

[17] (2020). Coronavirus devastates Detroit police, from the chief on down. The New York Times.

[18] Martinez N (2020). Shootings in Louisville up by more than 150 percent during coronavirus outbreak.

[19] Bates J (2020). Gun violence continues in Chicago amid stay-at-home orders. Time.

[20] Newall M, Palmer C, Purcell D (2020). Even the coronavirus can’t slow Philly’s gun violence.

[21] (2020). Philadelphia has a gun violence epidemic. What if it were treated like a contagious disease?.

[22] City of Philadelphia Office of the Mayor Department of Public Health (2020). Emergency Order Temporarily Prohibiting Operation of Non-Essential Businesses and Congregation of Persons to Prevent the Spread of 2019 Novel Coronavirus (COVID-19).

[23] Reed JA, Smith RS, Helmer SD (2003). Rates of unemployment and penetrating trauma are correlated. South Med J.

[24] Kim D (2019). Social determinants of health in relation to firearm-related homicides in the United States: a nationwide multilevel cross-sectional study. PLoS Med.

[25] Pew Research Center NW 1615 L. St, Suite 800 Washington, Inquiries D 20036 USA202-419-4300 | M-8578562| F-4194372| M (2020). Unemployment rose higher in three months of COVID-19 than it did in two years of the Great Recession.

[26] ABC News (2020). “Extreme inequality was the preexisting condition”: How COVID-19 widened America’s wealth gap.

[27] Gupta DB, Chun Y, Lee H (2020). Housing hardships reach unprecedented heights during the COVID-19 pandemic.

[28] Beheshti N (2020). 10 Eye-opening statistics on the mental health impact of the coronavirus pandemic.

[29] Everytown Research & Policy (2020). Gun violence and COVID-19.

[30] Temple University Hospital (2019). Temple Health University Hospital Community Health Needs Assessment.

[31] The New York Times (2020). Minority workers who lagged in a boom are hit hard in a bust. The New York Times.

[32] Bazemore A, Petterson S, Kushel M (2019). Adults with housing insecurity have worse access to primary and preventive care. J Am Board Fam Med.

[33] National Institute of Mental Health (2020). A new look at racial/ethnic differences in mental health service use among adults.

[34] Pew Research Center (2013). Blacks suffer disproportionate share of firearm homicide deaths.

[35] Oppel RA, Gebeloff R, Lai R (2020). The fullest look yet at the racial inequity of the coronavirus. The New York Times.

